# Superoxide- and semiquinone-linked activation of molecular hydrogen in metal-catalyst-free solution

**DOI:** 10.3389/fmolb.2025.1680812

**Published:** 2025-10-21

**Authors:** Toru Ishibashi, Enjuro Harunari, Genki Ishihara, Tetsushi Niiyama, Mami Noda-Urata, Nobuaki Komori

**Affiliations:** 1 Anicom Specialty Medical Institute (ASM), Tokyo, Japan; 2 Imabayashi Meiseikai Orthopedic Hospital, Kagoshima, Japan; 3 Biotechnology Research Center and Department of Biotechnology, Toyama Prefectural University, Toyama, Japan; 4 12 Pharmacy Inc., Tokyo, Japan; 5 Laboratory of Pathophysiology, Graduate School of Pharmaceutical Sciences, Kyushu University, Fukuoka, Japan

**Keywords:** molecular hydrogen, semiquinone, superoxide, quantum biology, quantum tunneling, H2 activation, reverse electron transport, mitochondrial complex I and III

## Abstract

The therapeutic effects of molecular hydrogen (H_2_), particularly in ischemia-reperfusion (I/R) injury and deleterious inflammation, have been increasingly attributed to its modulation of redox balance. However, the precise molecular mechanisms underlying H_2_-medated redox modulation, particularly in mitochondrial reverse electron transfer (RET)-driven superoxide (O_2_•^-^) generation, remain unclear. Here we show that under membrane-less in-solution conditions, H_2_ modulates O_2_•^-^ kinetics in ways consistent with a tunneling-assisted electron transfer involving semiquinone radicals (Q•^-^), without catalytic metals or hydrogenases. Using enzymatic (xanthine oxidase/hypoxanthine; XO/Hx) and non-enzymatic (potassium superoxide; KO_2_) systems combined with the O_2_•^-^-specific chemiluminescent probe, 2-methyl-6-p-methoxyethynyl-imidazopyrazinone (MPEC), we observed bell-shaped and U-shaped O_2_•^-^ kinetics as a function of H_2_. In Q-free assays, O_2_•^-^ appeared to activate H_2_, yielding a clear bell-shaped kinetic profile compatible with tunneling-assisted electron transfer from H_2_ to O_2_•^-^. When Q was present, distinct U-shaped profiles emerged, consistent with Q•^-^-mediated electron buffering followed by H_2_ activation. Electron spin resonance (ESR) radical scavenging experiments and quantitative high-performance liquid chromatography (HPLC) analyses confirmed transient semiquinone-mediated redox cycling leading to the formation of ubiquinol (QH_2_). Collectively, these in-solution data support a metal-free pathway for H_2_ participation in Q redox cycling that is compatible with tunneling-assisted electron transfer under defined *in vitro* conditions. These findings demonstrate the chemical feasibility of H_2_-driven Q reduction in-solution; the *in vivo* relevance remains to be determined.

## Introduction

H_2_ is attracting considerable attention not only a carbon-free energy carrier but also as a biologically active gas with therapeutic potential, particularly in pathological contexts involving mitochondrial dysfunction, inflammation, and I/R injury (Ohsawa et al., 2007; Xie et al., 2023; Ishibashi, 2019). Landmark work by Osawa and colleagues proposed that the therapeutic efficacy of H_2_ arises from its selective scavenging of hydroxyl radicals (•OH) in I/R injury (Ohsawa et al., 2007). However, subsequent experimental and theoretical analyses revealed that this •OH-scavenging mechanism cannot fully account for the observed protective effects. Notably, under physiological conditions, highly reactive •OH are immediately neutralized by surrounding cellular components such as amino acids and nucleic acids. Furthermore, O_2_•^-^ and hydrogen peroxide (H_2_O_2_), both continuously generated precursors of •OH, react with •OH much faster than H_2_ (Ishibashi, 2019; Halliwell and Gutteridge, 2015; Buxton et al., 1988). Considering the low reaction rates of H_2_ with •OH, the precise molecular mechanisms underlying H_2_-mediated biological effects remain unclear.

Biological utilization of H_2_ typically occurs through specialized hydrogenase enzymes found in certain prokaryotes and archaea (Greening et al., 2024). These hydrogenases contain transition-metal cofactors (Ni and/or Fe) within their catalytic center, structurally analogous to the Q binding site (Q-chamber) of mitochondrial NADH ubiquinone oxidoreductase (Complex I), and eukaryotic organisms, such as animals, plants, and yeast, have lost hydrogenases during mitochondrial evolution (Efremov and Sazanov, 2012; Schut et al., 2016). Consequently, any biological effects exerted by H_2_ in eukaryotes must involve alternative, catalytic metal independent mechanisms. We previously hypothesized that mitochondria might activate H_2_ at Q-chamber in Complex I. Energy converting electron transport reactions in Complex I generates highly reactive semiquinone intermediates, which exist in several one-electron reduced forms, including the anionic semiquinone radical (Q•^-^), the neutral semiquinone radical (QH•), and the anionic, deprotonated hydroquinone (QH^−^) (Murphy and Hartley, 2018). Among these species, Q•^-^ is the most reactive and is the only form that exchanges electrons with the O_2_/O_2_•^-^ couple under physiological conditions (Cape et al., 2006; Crofts et al., 1999). In mitochondrial electron transport, especially under conditions of RET, excessive generation of Q•^-^ leads to electron leakage to O_2_, triggering transient spikes of O_2_•^-^ production (O_2_•^-^ burst) (Hunte et al., 2010; Kussmaul and Hirst, 2006; Robb et al., 2018; Sorby-Adams et al., 2024). Given these characteristics, we hypothesized that Q•^-^ could serve as transient electron acceptors capable of activating H_2_ through a pathway distinct from the metal-catalyzed splitting of H_2_ observed in hydrogenases.

To test these hypotheses, we established a simplified *in vitro* experimental system using both enzymatic (XO/Hx) and chemical (KO_2_) methods for generating O_2_•^-^. We focused on the redox equilibrium between O_2_•^-^ and Q, and examined how H_2_ intervenes this equilibrium by quantifying O_2_•^-^ production. This approach allowed us to directly monitor the kinetics of reactions involving H_2_, O_2_•^-^, and Q without the complexity introduced by mitochondria or additional enzymatic processes beyond the initial O_2_•^-^ generation. Our experimental results suggest three distinct pathways of H_2_ activation. (1) Q•^-^-mediated tunneling. (2) direct tunneling-mediated by O_2_•^-^. (3) Q•^-^-mediated H_2_ activation independent of tunneling. These three pathways were inferred from kinetic profiles that align precisely with Marcus electron transfer theory (Marcus, 1993), particularly exhibiting inverted region behavior in which increasing the driving force paradoxically reduces the reaction rate, thus underscoring the quantum mechanical nature of these electron transfer reactions. Complementary analyses using ESR spectroscopy and quantitative HPLC analyses provided further insight into the three mechanisms above. These findings shed light on a previously unrecognized dual-mode mechanism of H_2_ redox activity.

Besides Q-centered pathways, porphyrin/heme-based mechanisms have been proposed as potential mediators of H_2_ chemistry (Jin et al., 2023). In particular, Fe-porphyrins have been reported to undergo H_2_-driven redox reactions and even to hydrogenate CO_2_ to CO under specific microenvironments, suggesting a porphyrin-linked route that could operate in biology. These ideas do not exclude a semiquinone-mediated route; rather, both may coexist and be operate in a context-dependent manner. In our in-solution study, we deliberately focused on Q•^-^ reactivity within a RET-relevant framework (complex I lacks heme), while acknowledging earlier discussions of the Q-cycle energy conversion in complex III where electron transfer between semiquinone and cytochrome *b*_L_ with Fe-heme centers play a pivotal role and could, in principle, also participate in H_2_ activation (including our own prior work) (Ishibashi, 2019).

Tunneling-mediated redox reactions have previously been observed exclusively within precisely tuned enzymatic environments, such as active sites in alcohol dehydrogenase or photosynthetic reaction centers (Klinman, 2006; Hay and Scrutton, 2012; Romero et al., 2014; Engel et al., 2007). However, activation of H_2_ under biologically relevant conditions without catalytic metals or specialized enzymes has remained unexplored. Notably, previous demonstrations of tunneling-assisted reactions under biological conditions have been restricted to enzyme-mediated environments. Our findings reveal, for the first time, the tunneling-driven H_2_ activation can occur through semiquinone radicals in aqueous solutions of physiological conditions without metal catalysts or specialized enzymatic frameworks, redefining quantum biochemical reaction boundaries in mitochondrial bioenergetics and it introduces potential industrial applications. Specifically, this catalytic-free H_2_ activation could dramatically reduce reliance on expensive metal catalysts in hydrogen energy technologies, facilitate environmentally benign syntheses in green chemistry, and potentially enable new catalyst-free strategies for pollutant remediation and sustainable chemical manufacturing.

## Materials and methods

### Materials and reagents

**FIGURE 1:**
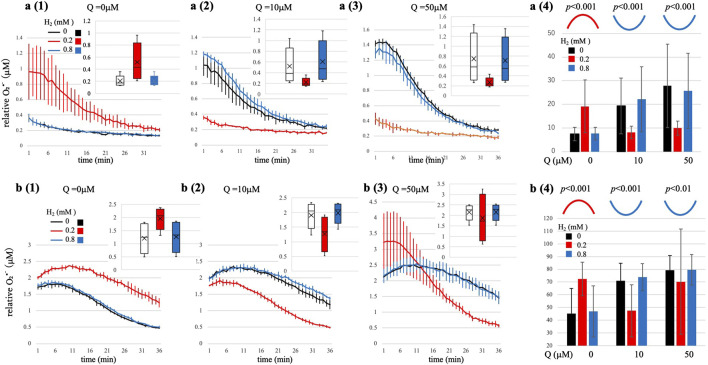
Time-resolved O_2_•^-^ generation in enzymatic (XO/Hx) systems under varying H_2_ and Q conditions. (a1-a3) Hx = 2 μM (b1-b3) Hx = 5 μM. Time course at Q = 0, 10, 50 μM, comparing H_2_ = 0 mM (black), 0.2 mM (red), and 0.8 mM (blue). Top-right insets: box plots: box: interquartile range, horizontal line: median: marker: mean; whiskers 5th-95th percentile. Exact ANOVA values are Supplementary Tables S3a,b. (a4, b4) Summary of Area Under the Curve (AUC, measured over 20 min, mean ± SD, n = 4) versus H_2_ showing bell-shaped curvature (red curve) at Q = 0 μM and U-shaped curvature (blue curves) at Q = 10 and 50 μM. Numerical *p*-values for curvature (quadratic term) are displayed in these summary panels. These kinetic profiles align with Marcus electron transfer theory (Marcus, 1993). Statistical note: Time-course traces (a1–a3, b1–b3) are descriptive only (mean ± SD; n = 4); inferential testing is performed on replicate-level AUC by ANOVA. Representative *p*-values for curvature (quadratic term; bell/U) are printed in a4 and b4; full statistics are provided in Supplementary Tables S3a,b.

All reagents were of analytical grade. Hx, XO, Q (Coenzyme Q10), QH_2_, and KO_2_ were obtained from Sigma-Aldrich (St. Louis, MO, United States of America). O_2_•^-^ was quantified using the fluorescent probe MPEC (ATTO Corporation, Osaka, Japan), according to manufacturer’s instructions. MPEC is a chemiluminescent probe that specifically reacts with O_2_•^-^, producing a fluorescent product. Compared to the commonly used 3, 7-dihydro-2-methyl-6-(4-methox-yphenol) imidazole pyrazin-3-one (MCLA), MPEC has lower background signals and higher specificity, providing accuracy for quantitative detection of O_2_•^-^ (Okutsu et al., 2012; Uchino et al., 2012; Kimoto et al., 1993). Hydrogen-saturated water (>8 ppm H_2_) was freshly prepared using a high-pressure H_2_ dissolving system (Trust 8.0 Hydrogen Water Generator, Trust Co. Ltd., Fukuoka, Japan). The concentration of H_2_ was verified using the methylene blue/colloidal-platinum colorimetric assay (Seo et al., 2012). To minimize uncertainty from degassing, all assays were initiated immediately after mixing and completed within 5–60 min. This time window was chosen *a priori* based on our published time-course data showing that dissolved H_2_ in water (5 ppm in the report) remains ∼78% of its initial value after 60 min (5.40 ± 0.12 mg/L immediately; 4.22 ± 0.15 mg/L at 1 h) (Ishibashi et al., 2012), implying ∼98% retention at 5 min (for Figure 4) and ∼86% at 36 min (for Figure 1) under a simple first-order loss model. Accordingly, H_2_ remains present at high fractional levels throughout our assays. Unless otherwise specified, all solutions were prepared in phosphate-buffered saline (PBS: 68.5 mM NaCl 1.35 mM KCl, 5.1 mM Na_2_HPO_4_, 0.88 mM KH_2_PO_4_, pH 7.4).

## Superoxide quantification by MPEC fluorescence assay

**FIGURE 2:**
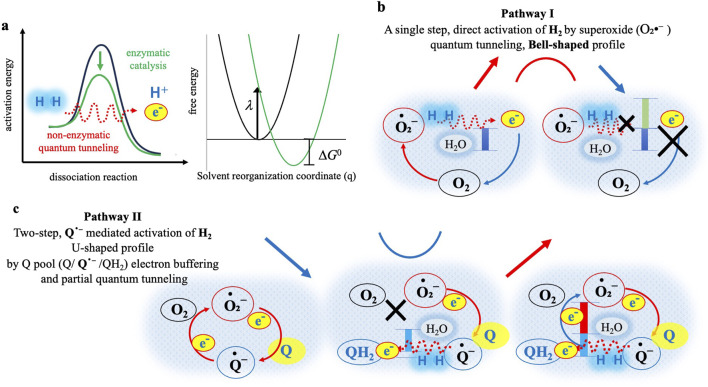
Proposed mechanisms underlying the bell-shaped (Pathway I) and U-shaped (Pathway II) kinetic profiles observed for O_2_•^-^ generation under varying H_2_ and Q conditions. Possible tunneling is depicted by red dotted line, and blue shading (dotted pale blue) around the red dashed arrow represents surrounding H_2_O molecules, indicating solvent reorganization (λ) as described by Marcus theory. **(a)** Left: Illustration of activation energy requirements for molecular hydrogen (H_2_) dissociation. The green curve represents classical enzymatic catalysis, typically facilitated by hydrogenase enzymes, reducing the activation barrier and enabling efficient dissociation into protons (H^+^) and electrons (e^−^). The red dotted line depicts non-enzymatic tunneling, allowing H_2_ dissociation without enzymes. Right: Direct electron tunneling from H_2_ to O_2_•^-^ or Q•^-^ in aqueous solvent. Inset: Marcus free-energy parabolas plotted against the solvent reorganization coordinate (q, dimensionless), showing λ (reorganization energy) and Δ*G* ^0^ (driving force). The calculated activation barrier (Δ*G* ^‡^) under Q-free conditions is very small (0.017 eV; see Supplementary Methods, Supplementary Equation 2) and therefore not drawn to scale, but noted in the text. **(b)** Pathway I (Q-free): Potential tunneling-mediated activation of H_2_ by O_2_•^-^ may produce a bell-shaped profile. Left: At optimal driving force (blue bar), tunneling may efficiently transfer electrons to O_2_, increasing O_2_•^-^ (red arrow). Right: At excessive H_2_ concentrations, tunneling could enter the Marcus inverted region (green bar), leading to reduced electron transfer efficiency (cross marks) and decreased O_2_•^-^ formation (blue arrow). **(c)** Pathway II (with Q): A two-step, Q•^-^ mediated activation of H_2_ leading to a U-shaped profile. Left: In the absence of H_2_, Q continuously buffers electrons from O_2_•^-^, sustaining baseline O_2_•^-^. Middle: At low H_2_, Q•^-^ could activate H_2_ directly or through tunneling (red dotted line) and reduce Q•^-^ to QH_2_ (blue bar), thereby cutting off O_2_/O_2_•^-^ recycling (cross mark) and lowering the amount of O_2_•^-^ (blue arrow). Right: At high H_2_ concentrations, the excess electrons from activated H_2_ may exceed the buffering capacity of the Q-pool, and the leaked electrons (red bar) resume the recycling of the O_2_•^-^ generation (red arrow), completing the right side of the U-shaped kinetic profile.

O_2_•^-^ production was measured in real time using the MPEC fluorescence assay. MPEC was included at 100 μM in all assays. The plate reader recorded fluorescence intensity (360 nm excitation/450 nm emission) every minute for a total of 15–60 min. Each condition was measured in four replicates. Reactions were carried out in 96-well black microplates (100 μL per well) at 30 °C and monitored on an EnSpire® Multimode Plate Reader (PerkinElmer). For enzymatic O_2_•^-^ generation, we used the Hx/XO system: typically, Hx (at 2, 5, or 200 μM as specified) and XO (0.01–0.02 U/mL) in PBS. In parallel, a non-enzymatic O_2_•^-^ source was prepared by dissolving KO_2_ in potassium phosphate buffer at final KO_2_ concentrations of 1 or 2.5 mM. Reaction mixtures were prepared with or without Q. Coenzyme Q10 (Q) has low aqueous solubility but highly soluble in organic solvents, with a reported solubility in DMF exceeding 10 mM (Kommuru et al., 1999; Bhagavan and Chopra, 2006). Thus, Q was freshly prepared as a 10 mM solution in DMF and diluted into assay buffer immediately prior to each experiment to minimize any solvent-related instability. To keep the final buffer composition (substrate, enzyme, chemical compounds, phosphate/salt content including DMF) identical across all conditions, the same volumes of buffer components and DMF were dispensed to every well, thus, only Q and H_2_ differed between groups. The final DMF concentration was 0.5% (v/v) in all assays with Q ≤ 50 μM except for 250 μM Q with 2.5% (v/v, Figure 2a(1–3)).

Fluorescence intensity data were initially recorded as arbitrary fluorescence units per minute (AFU/min). Because fluorescence intensity measurements can vary substantially depending on instrument sensitivity, optical configuration, and experimental conditions, direct absolute quantification of O_2_•^-^ concentrations were impractical. To facilitate comparative kinetic analyses, we converted AFU/min values into relative O_2_•^-^ concentrations (μM) based on previously reported calibration data for MPEC chemiluminescent probes (Hansel et al., 2019; Yamazaki et al., 1999; Shimomura et al., 1998). Among these reports in which the fluorescence intensities range broadly from approximately 10^4^ to 10^6^ counts per μM, we defined approximately 10,000 AFU as equivalent to 1 μM of relative O_2_•^-^ concentration based on the calibration data from enzymatic systems closely similar to those in our current study (Shimomura et al., 1998). Thus, all reported O_2_•^-^ concentrations in this study are relative measures intended for comparative analysis, rather than absolute quantification. This relative concentration scale aligns well with commonly reported intracellular steady-state O_2_•^-^ concentrations, which typically range from low nanomolar to sub-micromolar levels in biological systems (Hansel et al., 2019; Yamazaki et al., 1999; Shimomura et al., 1998). No exogenous superoxide dismutase (SOD) was added during these assays in order to observe the native kinetics of O_2_•^-^; we note this as a limitation (see Study Limitations) but rely on specificity of MPEC to ensure the signal represents O_2_•^-^ (Shimomura et al., 1998).

### ESR analysis of radical scavenging activity using DPPH

To probe the formation of radical intermediates (such as semiquinone radicals including Q•^-^) and the involvement of H_2_ in redox cycling, we employed electron spin resonance (ESR) spectroscopy. Because the Q•^-^ has a sub-millisecond lifetime in aqueous solution, making direct ESR detection infeasible, we utilized DPPH as a stable radical probe to detect radial-scavenging activity indirectly. Reduction (scavenging) of the DPPH radical under different reaction conditions serves as an indirect indicator of radical generation and quenching capacity. Reaction mixtures for ESR were prepared in phosphate buffer (pH7.4) containing 200 µM Hx, 0.02 U/mL XO, 250 µM Q, and 200 µM DPPH. After 5 min incubation at room temperature (with or without dissolved H_2_ as specified), samples were loaded into quartz capillary tubes for ESR measurements. ESR spectra were recorded on a Bruker EMX nano spectrometer under the following parameters: microwave frequency ∼9.85 GHz, modulation frequency 100 kHz, sweep width 100 G, and time constant 40.96 m. Each condition was measured in triplicate, and the spectra were averaged, and the intensity of the DPPH ESR signal was quantified. Diminution of the DPPH signal indicates net radical scavenging activity in the sample, reflecting the presence of strong reducing agents (e.g., QH_2_ or other radical species capable of donating electrons to DPPH).

### HPLC quantification of QH_2_ formation

Formation of QH_2_ was directly measured by HPLC to confirm the participate of H_2_ and Q in redox cycle. Reactions (500 µL volume) contained KO_2_ (2.5 mM) as a chemical superoxide source, 50 µM Q and 1 mM potassium phosphate buffer (1 mM, pH 6.0, chosen to favor QH_2_ stability). After incubation for 5 min at room temperature (with or without H_2_), reactions were quenched by adding formic acid to a final concentration of 2% (v/v). Quenched samples were immediately analyzed using a Waters Alliance HPLC system equipped with a reverse-phase C18 column (Nacalai Tesque AR-II, 4.6 × 100 mm). The mobile phase was an isocratic mixture of methanol and ethanol (1:1, v/v) delivered at a flow rate of 1.0 mL/min. Elution of Q and QH_2_ was monitored by UV absorbance at 290 nm. Peaks corresponding Q and QH_2_ were identified by their characteristic retention times, confirmed using authentic standards, and quantified by peak area integration. Each condition (+H_2_ vs. -H_2_) was measured in 27 times replicates to ensure robust statistics validity due to the expected variability inherent in radical-based reactions. The percent conversion of Q to QH_2_ was calculated for each replicate.

### Statistical analysis and nonlinear model fitting

All statistical analyses were performed using Python 3.11 using standard scientific libraries (NumPy, SciPy, pandas) and statistical packages Statsmodels (for analysis of variance (ANOVA) and regression analyses). Time-course fluorescence data of O_2_•^-^ were first integrated area under the curve (AUC) to quantify total O_2_•^-^ production under each condition. Two-way ANOVA was used to evaluate the effects of Q and H_2_ concentration on both initial O_2_•^-^ generation rates and AUCs. One-way ANOVA was conducted for single-factor comparisons where appropriate with a significance threshold α = 0.05. Quadratic polynomial regression analysis (y = ax^2^ + bx + c) were conducted to characterize non-linear AUC trends in H_2_-dependent O_2_•^-^ production. A significantly negative quadratic coefficient (a < 0) indicated a bell-shaped dependence (maximum rate at intermediate H_2_), whereas a significantly positive coefficient (a > 0) indicated a U-shaped dependence (minimum rate at intermediate H_2_). Regressions were fit ordinary least squares, and curvature significance (deviation from linearity) was evaluated by reporting *p*-values. Levels of QH_2_ formation in paired samples (±H_2_) from each experimental day were compared by paired *t*-tests (validated by repeated-measured ANOVA), and variability was assessed via coefficient of variation (CV%).

To interpret the experimental kinetic data within the framework of electron transfer theory, the H_2_-dependent O_2_•^-^ production was analyzed using Marcus’ outer-sphere electron transfer model (Gray and Winkler, 2005). The Marcus rate equation is given by:

$$k=A\exp\left[-\frac{\left({\Delta G}^{0}{\left.+\lambda \right)}^{2}\right.}{4\lambda k_{B}T}\right]$$

Where *λ* represents the reorganization energy (eV), Δ*G* ^0^ is the Gibbs free energy change for the reaction (eV), *k*_*B*_ is Boltzmann’s constant, and *T* is absolute temperature. Δ*G* ^0^ values for relevant reactions were estimated using standard midpoint potentials converted to the Δ*G* ^0^ in eV: H_2_/H^+^ ≈ −0.41 V; Q/Q•^-^ ≈ 0 V; QH_2_/Q ≈ +0.065 V; O_2_/O_2_•^-^ ≈ −0.16 to −0.33 V, depending on pH. For systems lacking Q, a single-step electron transfer (H_2_ to O_2_) was modeled, whereas for Q containing systems, a two-step sequential transfer (H_2_ → Q•^-^ followed by Q•^-^ → QH_2_ or O_2_•^-^) was assumed. Nonlinear least-squares fitting (SciPy’s curve_fit) was performed to determine optimal values of *λ* and amplitude prefactor, *A*, for each condition (see Supplementary Table S1). The fitted *λ* values (0.120–0.180 eV) were used to determine reaction regimes (normal versus inverted) relative to the driving force. The amplitude parameter *A*, which corresponds to the preexponential factor in Marcus theory and reflects electronic coupling and collision frequency between donor and acceptor (Gray and Winkler, 2005), was treated as an empirical scaling factor. While *A* was included in the fits for completeness, kinetic behavior comparisons were primarily based on the fitted *λ* values. For example, higher Q concentrations typically increased the fitted *A* value, indicating enhanced electron coupling or collision efficiency facilitated by the redox mediator, though the qualitative kinetic behaviors were principally delineated by *λ*. Statistical significance levels and fit parameters are reported throughout, and errors are provided as standard deviations (SD).

## Results and discussion

### H_2_ modulates the redox cycling between O_2_•^-^ and Q

We first established a baseline by examining how the presence of Q affects O_2_•^-^ levels in the XO/Hx enzymatic system in the absence of H_2_. Q can accept an electron from O_2_•^-^ to form Q•^-^, as described by the fundamental equilibrium:

$$O_{2}\cdot^{-}+Q\;⇄\;O_{2}+Q\;\cdot^{-}$$ (1)

The forward reaction (O_2_•^-^ donating an electron to Q) is kinetically highly favored, with reported rate constants on the order of 10^6^ ∼ 10^8^ M^-1^s^-1^, whereas the reverse reaction (Q•^-^ returning an electron to O_2_) is 3-4 orders of magnitude slower (10^3^ ∼ 10^6^ M^-1^s^-1^) (Song et al., 2008; Maroz et al., 2009). This pronounced kinetic asymmetry means that electrons leaking from O_2_•^-^ become transiently trapped as Q•^-^, leading to the accumulating the Q•^-^ and consequently stabilizing O_2_•^-^ levels. Indeed, our experiments showed that increasing Q concentration resulted in higher steady-state O_2_•^-^ signals. For instance, at a low Hx concentration (2 μM), the mean initial O_2_•^-^ production rate (±SD) increased from 0.25 ± 0.05 μM/min (relative concentration, calculated from fluorescence counts (AFU/min) as described in Methods) at 0 μM Q to 1.22 ± 0.21 μM/min at 50 μM Q. At a higher Hx concentration (5 μM), the rate increased from 1.73 ± 0.07 μM/min (0 μM Q) to 2.38 ± 0.11 μM/min (50 μM Q). These increases were statistically significant (one-way ANOVA, *p* < 0.01; see Supplementary Tables S2a,b), confirming that Q effectively traps electrons as Q•^-^, leading to a higher steady-state concentration of detectable O_2_•^-^. This behavior is consistent with reaction (1), wherein Q scavenges electrons from O_2_•^-^, thereby preventing its immediate dismutation and elevating detectable O_2_•^-^ concentrations.

### Nonlinear modulations of the redox cycling between O_2_•^-^ and Q by H_2_

Having validated that our system can generate and detect Q•^-^-mediated changes in O_2_•^-^, we next investigated the influence of H_2_. We introduced varying concentrations of H_2_ (0–0.8 mM) into reactions at different Q concentrations and monitored the amount of O_2_•^-^ in real time using MPEC fluorescence. At low substrate concentrations (Hx = 2 μM and 5 μM), we observed distinct non-linear kinetic profiles in response to varying H_2_ concentrations depending on the presence or absence of Q. Without Q, the system exhibited a bell-shaped kinetic profile, whereas in the presence of Q, a clear U-shaped kinetic profile emerged. These differing profiles strongly suggest that the underlying mechanisms of H_2_ dissociation differ mechanistically based on Q availability, as further discussed below.

Figure 1 summarizes the results obtained under two representative substrate conditions (Hx = 2 μM and 5 μM). In both cases, H_2_ exhibited a biphasic effect on O_2_•^-^ levels dependent upon the presence of Q. To quantitatively characterize these non-linear kinetic responses, we performed quadratic regression analyses (y = ax^2^ + bx + c) on the integrated O_2_•^-^ levels (AUC) versus H_2_ concentration at each fixed Q concentration (see Supplementary Tables S3a,b for detailed statistical results).

1. At Q = 0 μM (Figures 1a(1),b(1); Hx = 2 μM and 5 μM, respectively): A significant bell-shaped curvature was observed (Hx = 2 μM: a = -30.7, *p* < 0.001; Hx = 5 μM: a = −40.3, *p* < 0.001; Figures 1a(4),b(4), respectively). At lower H_2_ concentrations (0.2 mM), O_2_•^-^ production was maximized, indicating an optimal concentration of H_2_. However, at higher H_2_ concentration (0.8 mM), the amount of O_2_•^-^ generation declined back to levels comparable to those without H_2_, clearly demonstrating inverted-region kinetic behavior at elevated H_2_ concentration.
2. At Q = 10 μM (Figures 1a(2),b(2); Hx = 2 μM and 5 μM, respectively): A significant U-shaped curvature emerged across the tested H_2_ concentrations (Hx = 2 μM: a = +68.0, *p* < 0.001; Hx = 5 μM: a = +29.7, *p* < 0.001, Figures 1a(4) and b(4), respectively). At 0 mM H_2_, O_2_•^-^ generation was near maximal. At lower H_2_ concentration (0.2 mM), O_2_•^-^ production significantly dropped to minimal levels, whereas, at higher H_2_ concentrations (0.8 mM), the amount of O_2_•^-^ increased again, returning to baseline levels comparable to those observed at 0 mM H_2_. This U-shaped response clearly differed from conditions without Q.
3. At Q = 50 μM (Figures 1a(3),b(3); Hx = 2 μM and 5 μM, respectively): Similar significant U-shaped curvature in AUC were observed (Hx = 2 μM: a = +29.8, *p* < 0.001; Hx = 5 μM; +9.49, *p* < 0.01; Figures 1a(4) and b(4), respectively). At Hx = 2 μM (Figure 1a(3)), O_2_•^-^ generation was maximal at 0 mM H_2_ and significantly reduced at lower H_2_ (0.2 mM), but increased again at higher H_2_ concentrations (0.8 mM), demonstrating a consistent U-shaped kinetic profile. Interestingly, At Hx = 5, (Figure 1b(3)), the kinetic behavior was more complex: At H_2_ concentrations of 0 and 0.8 mM (black lines), the amount of O_2_•^-^ was initially low and similar, whereas at an 0.2 mM H_2_ (a red line), an unusual drastic profile was observed: initially high, but declining below the levels observed at 0 and 0.8 mM H_2_ midway through the measurement. This reversal indicates dynamic changes in the redox state during the reaction, possibly reflecting an intermediate-driven mechanism or altered electron distribution between Q•^-^ and O_2_•^-^ pathways. Further investigation is warranted to clarify this distinctive kinetic behavior.

These kinetic profiles under conditions of low substrate availability for O_2_• production (Hx = 2 μM and 5 μM) clearly indicate that the influence of H_2_ on O_2_•^-^ generation differs markedly depending on whether Q is present or absent. In the absence of Q, a distinct bell-shaped kinetic profile emerged, indicative of tunneling-mediated electron transfer from H_2_ to O_2_, consistent with Marcus inverted-region kinetics. In contrast, in the presence of abundant Q (10 μM or 50 μM), a reproducible U-shaped kinetic profile was observed. Note that due to technical limitations in reliably dissolving higher concentrations of H_2_ (>0.8 mM), we could not explore whether the U-shaped profiles might exhibit inverted-region kinetics at even at higher H_2_ concentrations in the presence of Q.

In the next sections, we discuss the mechanistic implications of these observations in detail, exploring tunneling as a plausible mechanism underling the bell-shaped kinetics in the Q-free system, and the dual electron-buffering and activating role of Q•^-^ in the presence of Q. Subsequently, we will present further experimental results (ESR and HPLC analyses) supporting these mechanistic interpretations, followed by the analysis of more complex kinetic behaviors observed under condition of higher substrate flux (Hx = 200 μM) and the enzyme-free, KO_2_-driven O_2_• generation system.

### Quantum tunneling-mediated oxidation of H_2_ by O_2_•^-^ (pathway I)

In our Q-free system, the O_2_•^-^ initially generated from Hx oxidation is proposed to activate H_2_ as schematically illustrated in Figure 2a, b (Pathway I). Under typical aqueous conditions without enzymatic or structural catalysis, such a direct electron transfer from H_2_ to O_2_ would be thermodynamically and kinetically improbable, given the high H-H bond dissociation energy of approximately 435 kJ/mol (Luo, 2007), and the spin-forbidden nature of the O_2_ reduction reaction (Valentine et al., 1998; Hayyan et al., 2016). Despite these intrinsic barriers, our experimental results clearly demonstrate measurable increase of O_2_•^-^, especially pronounced at lower H_2_ concentration (0.2 mM, red lines in Figures 1a(1),b(1)). These observations strongly suggest the occurrence of electron transfer under conditions where classical reaction pathways appear unlikely.

Integrated O_2_•^-^ production levels (AUC; Figures 1a(4),b(4)) exhibited a distinct bell-shaped kinetic profile, with a peak around 0.2 mM H_2_ and reduced production at a higher concentration (0.8 mM). This observed kinetic behavior aligns remarkably well with the parabolic relationship between electron transfer rate and driving force predicted by Marcus electron transfer theory (Marcus and Sutin, 1985). According to Marcus theory, electron transfer rate initially increases with greater driving force (Marcus normal region), but beyond an optimal point, further increases in driving force could paradoxically suppress electron transfer rate, which is a phenomenon known as the Marcus inverted region. Indeed, kinetic modeling of our data provided a calculated reorganization energy *λ* of 0.147 eV under Q-free conditions, consistent with an optimal driving force for electron transfer around 0.2 mM H_2_, presenting Marcus normal region. At a higher H_2_ concentration (0.8 mM), the model indicates a shift in the Marcus inverted region, explaining the observed paradoxical reduction in electron transfer efficiency generating O_2_•^-^ despite the possible increasing of the driving force (Figure 2b).

In Marcus theory, electron transfer rates are critically dependent on solvent reorganization energy (*λ*), which represents the energy required for the surrounding medium (e.g., solvent dipoles and water molecules) to reorganize in response to the change in charge distribution following electron transfer. This framework was established in foundational work by Marcus and co-workers (Marcus and Sutin, 1985) and has been further developed in experimental/theoretical and simulation studies of biological ET (Barthel et al., 2001; Blumberger, 2015). In our model (Figure 2a), the driving force Δ*G* ^0^ and reorganization energy *λ* are explicitly considered, and very small activation barrier Δ*G* ^‡^ is calculated under Q-free conditions (0.017 eV; see Supplementary Methods, Supplementary Equation 2). Thus, our approach follows the methodology used in these theoretical frameworks. Considering the absence of catalytic enzymes or organized molecular environments in our experimental setup, effective electron transfer would require molecules to approach each other at near van der Waals distance, typically within a few angstroms between donor and acceptor molecules with highly specific orientations (Marcus and Sutin, 1985; Nagel and Klinman, 2006; Klinman and Kohen, 2013). Such precise interactions are expected to be rare. Nonetheless, our clear experimental detection of electron transfer strongly supports tunneling at the most plausible explanation for overcoming these significant kinetic and thermodynamic barriers.

### Mechanistic details of Q-Mediated two-step electron transfer (pathway II)

In Q-mediated Pathway II (Figure 2c), the presence of Q fundamentally alters the mechanism. Crucially, O_2_•^-^ initially generated by the oxidation of Hx donates an electron to Q, forming Q•^-^ (Equation 1). The electron stored in Q•^-^ acts as an electron buffer, transiently stabilizing O_2_•^−^levels, resulting in maximal initial O_2_•^-^ concentrations early in the reaction (black lines in Figures 1a(2,3),b(2)). Upon addition of low dose of H_2_ (0.2 mM), O_2_•^-^ levels distinctly decreased (red lines in Figures 1a(2,3),b(2)). This result clearly indicates that Q•^-^ directly interact with and activates H_2_, accepting electrons from H_2_ to form reduced QH_2_. As Q•^-^ become reduced to QH_2_, the electron buffering capacity of Q-pool diminishes, resulting in the decrease of O_2_•^-^ formation. This observation represents the first experimental demonstration that H_2_ can actively intervene in electron transfer equilibrium between O_2_•^-^ and Q.

At higher H_2_ concentrations (0.8 mM; blue lines in Figures 1a(2,3),b(2)), this trend reversed, and O_2_•^-^ levels returned to baseline values observed without H_2_. This restoration of O_2_•^-^ is likely due to saturation of the electron-buffering capacity of Q/Q•^-^ pool. Under these conditions, excess electrons from H_2_ can exceed the electron accommodated capacity of the Q•^-^, redirecting electrons back to O_2_, thus restoring O_2_•^-^ generation. However, it is important to note that increased formation of QH_2_ under high H_2_ conditions may also scavenge O_2_•^-^, partially offsetting this restoration, thereby representing the U-shaped kinetic profile as shown in Figure 2c.

While the U-shaped behavior clearly observed at these conditions strongly supports a two-step electron transfer mechanism mediated by Q•^-^, it remains possible that Marcus inverted-region behavior could emerge at even higher concentrations of dissolved H_2_ (e.g., 1.6 mM). Exploring such high H_2_ concentrations would require improvements in experimental conditions to reliably dissolve higher concentrations of H_2_. Due to practical limitations, we restricted our experiments to a maximum of 0.8 mM H_2_. Nevertheless, possible Marcus inverted-lesion kinetics are suggested by data obtained under other experimental conditions (higher substrate flux conditions with Hx = 200 μM, and KO_2_ = 1 mM), discussed in subsequent sections.

In summary, the Q-mediated Pathway II illustrates a distinct two-step mechanism in which Q•^-^ activate H_2_, supplying electrons from H_2_ to form QH_2_. This electron-buffering function of Q-pool initially suppresses O_2_•^-^ generation at lower H_2_ concentrations, resulting in the characteristic U-shaped kinetic profile. This mechanism clearly differs fundamentally from Pathway I, where Q is absent.

### Superoxide kinetics at high substrate flux

**FIGURE 3:**
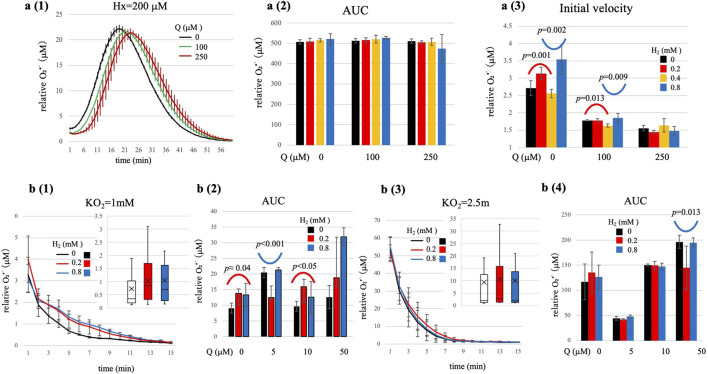
O_2_•^-^ generation kinetics under high-substrate flux conditions (Hx = 200 μM, a1-a3), and O_2_•^-^ generation by chemical system (KO_2_, b1-b4). (a1) Representative time-course showing slower and sustained O_2_•^-^ generation in the absence of H_2_. (a2) Total O_2_•^-^ production (AUC; mean ± SD, n = 4) did not vary significantly with H_2_ or Q. (a3) Initial O_2_•^-^ generation rates (first 3 min, mean ± SD, n = 4) showed significant non-linear dependencies on both H_2_ and Q concentrations; statistics in Supplementary Table S4. At Q = 0 μM and 100 μM, significant bell-shaped (lower H_2_ range) and U-shaped (higher H_2_ range) curves were observed. At Q = 250 μM, similar but statistically non-significant trends were observed. (b1-b2) KO_2_ = 1 mM; (b3-b4) KO_2_ = 2.5 mM. Time courses with H_2_ = 0, 0.2, 0.8 mM in the absence of Q. Top-right insets: box plots: box: interquartile range, horizontal line: median: marker: mean; whiskers 5th-95th percentile. Exact ANOVA values are Supplementary Tables S5a,b. At KO_2_ = 1 mM, significant bell-shaped (Q = 0, 10 μM) and U-shaped (Q = 5 μM) dependencies are observed. At KO_2_ = 2.5 mM, H_2_ modulation was largely masked, with only a minor U-shape at Q = 50 μM (*p*-values shown in panels). Statistical note: Time-course traces are descriptive only (mean ± SD; n = 4). For Hx = 200 μM, inference uses initial velocity (1–3 min) analyzed by ANOVA (see a3; Supplementary Table S4). For KO_2_ panels, inference uses AUC analyzed by ANOVA (see b2 and b4; Supplementary Tables S5a,b). Representative *p*-values are printed in the summary panels.

Next, we examined whether the modulation of O_2_•^-^ by H_2_ observed under low substrate flux conditions, persists under conditions of much higher redox flux. For this purpose, we significantly increased the substrate (Hx) concentration to 200 μM for XO). Under these conditions, XO activity becomes saturated, resulting in slower but sustained O_2_•^-^ generation. Notably, total O_2_•^-^ accumulation (AUC) was consistently high and showed no significant variation with either H_2_ and Q concentration. A two-way ANOVA revealed neither significant main effects nor a significant interaction between H_2_ and Q (Figure 3a(1–2)). Thus, under conditions of prolonged and saturating substrate supply, H_2_ did not significantly alter overall O_2_•^-^ production.

However, careful analysis of initial O_2_•^-^ production velocities (measured within the first 1–3 min) revealed subtle yet distinct non-linear effects of both H_2_ and Q concentrations (*p* < 0.001, see Supplementary Table S4). Specifically, segmented quadratic regression analyses identified notable inverted kinetic patterns as follows:

1. At Q = 0 μM: A clear and significant bell-shaped dependence on H_2_ at lower concentration ranges (0–0.4 mM; quadratic coefficient a = −12.4, *p* = 0.001), transitioning into a significant U-shaped dependence at higher H_2_ range (0.2–0.8 mM; a = + 8.89, *p* = 0.002).
2. At Q = 100 μM: Similar trends, although less pronounced, were evident: a significant bell-shaped dependence in the lower H_2_ concentration range (0–0.4 mM; a = −1.93, *p* = 0.013), and a significant U-shape dependence at higher H_2_ concentrations (0.2–0.8; a = + 2.20, *p* = 0.009).
3. At Q = 250 μM: While similar curvature patterns were quantitatively evident (U-shaped at lower H_2_, bell-shaped at higher H_2_), statistical significance was not reached (low H_2_ range, *p* ≈ 0.08; high H_2_ range, *p* ≈ 0.06). This suggests that optimal or inverted kinetic regimes may occur beyond the tested H_2_ concentrations at such high Q levels.

These findings demonstrate that even under conditions of high substrate flex, where O_2_•^-^ production is unaffected by H_2_ due to excess substrate, initial electron transfer kinetic remain sensitive to H_2_ modulation. The coexistence of bell-shaped and U-shaped kinetic profiles within segmented H_2_ concentration ranges strongly suggests a complex Marcus-type relationship between electron transfer rates and driving force. Specifically, as H_2_ concentration increases, the reaction initially approaches an optimal driving force region, then shifts into the Marcus inverted region (bell-shaped profile), and subsequently enters in U-shaped profile, reflecting changes in electron transfer efficiency with different mechanisms. Otherwise, the optimal H_2_ concentrations for electron transfer likely may lie near or slightly beyond our tested range, resulting in partial or segmented kinetic profiles within our analyzed concentration ranges.

### The tunneling-modulated behavior of H_2_ under chemical O_2_•^-^ generation

To confirms that the previously described H_2_ modulation of O_2_•^-^ is not unique to the enzymatic (XO/Hx) system, we next examined a purely chemical source of O_2_•^-^, KO_2_ in aqueous buffer. KO_2_ generates O_2_•^-^ spontaneously upon hydration, allowing us to test whether the observed H_2_ effects persist independently of enzymatic processes. Results under moderate (KO_2_ = 1 mM) and high oxidative flux condition (KO_2_ = 2.5 mM) are summarized in Figure 3b. Under moderate oxidative flux conditions (KO_2_ = 1 mM), two-way ANOVA indicated significant main effects and interactions for both H_2_ and Q concentrations on total O_2_•^-^ production (AUC, *p* < 0.001, see Supplementary Table S5a). Specifically, we observed distinct non-linear kinetic profiles analogous to the enzymatic system:

1. At lower Q concentrations (0 or 10 μM): H_2_ induced significant bell-shaped AUC profiles (e.g., at Q = 0 μM, a = -30.7, *p* ≈ 0.04; at Q = 10 μM, a = −45.9, *p* < 0.05). These profiles align closely with Marcus theory, suggesting tunneling-mediated electron transfer mechanisms at optimal H_2_ concentrations.
2. At intermediate Q concentrations (5 μM): A clear and statistically significant U-shaped kinetic profile was observed (a = +68.0, *p* < 0.001). This indicates minimal O_2_•^-^ accumulation at lower H_2_ concentrations, consistent with a two-step electron buffering mechanism mediated by Q•^-^, as described earlier.
3. At high Q concentration (50 μM): H_2_ dependence on O_2_•^-^ production was essentially flat (no significant curvature detected), suggesting effective electron buffering by the abundant Q-pool. Thus, under these conditions, the modulatory influence of H_2_ was limited.

In contrast, under the higher oxidative flux condition (KO_2_ = 2.5 mM), the modulatory effects of H_2_ became less pronounced (Figure 3b(3–4) , see Supplementary Table S5b). Here, O_2_•^-^ levels rapidly rose to very high concentrations, overwhelming any measurable modulatory influence of H_2_ within the timeframe of the experiment. Although Q concentration continued to significantly influence total O_2_•^-^ accumulation by electron trapping (two-way ANOVA, *p* < 10^–10^, Supplementary Table S5b), H_2_ addition did not yield statistically significant changes in total O_2_•^-^ levels. Only at Q = 50 μM was a minor but significant U-shaped curvature detected (*p* = 0.013). This diminished modulation by H_2_ likely reflects an extremely narrow kinetic window or indicates that effective H_2_ modulation occurs at concentrations higher than those tested, potentially masked by rapid redox cycling processes (e.g., rapid re-oxidation of QH_2_ to Q and subsequent O_2_•^-^ generation).

Taken together, these enzymatic (presented the former sections and chemical experiments consistently support a dual-modulatory role for H_2_. Under relatively mild oxidative conditions (both enzymatic and chemical), H_2_ can modulates O_2_•^-^ levels, generating clear bell-shaped or U-shaped kinetic profiles consistent with Marcus electron transfer theory. Under extreme oxidative conditions (e.g., with KO_2_ = 2.5 mM), the O_2_•^-^ concentrations far exceed physiologically relevant levels which is typically within the low micromolar range and transiently reaching up to tens of micromolar during bursts (Murphy, 2009), and the modulatory effect of H_2_ become less evident. The consistency between enzymatic and non-enzymatic systems strongly suggests that these observed phenomena reflect intrinsic molecular interactions among H_2_, O_2_•^-^, and Q, rather than an enzyme-specific artifacts.

### Indirect ESR evidence supporting semiquinone radical (Q•^-^)-mediated H_2_ activation

To experimentally test the hypothesis that semiquinone radical (Q•^-^) mediate the H_2_-dependent modulation of redox reactions, we employed ESR spectroscopy to indirectly detect semiquinone radicals or their downstream reduction product, QH_2_. Direct ESR detection of Q•^-^ was impractical due to their extremely short lifetime in aqueous solution and their coexistence with abundant O_2_•^-^ radicals. Therefore, we indirectly assessed radical formation by measuring radical scavenging activity against a stable reference radical, DPPH, under conditions optimized to clearly demonstrate H_2_-dependent redox effects where moderate oxidative stress and sufficient Q concentrations known to yield U-shaped kinetic profiles.

**FIGURE 4:**
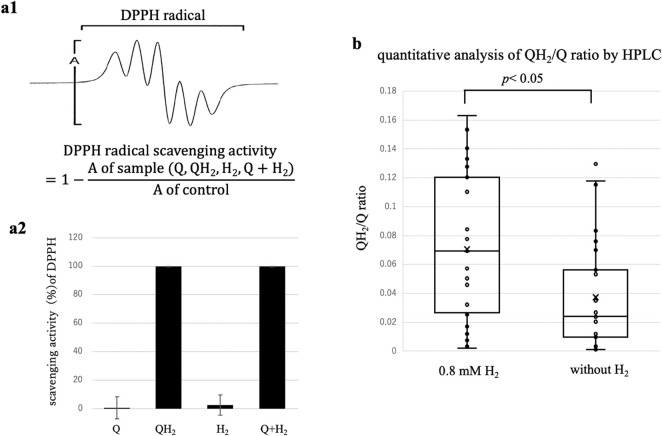
Evidence supporting semiquinone radical-mediated H_2_ activation and QH_2_ formation. **(a)** ESR analysis of radical scavenging activity using the stable radical DPPH: (a1) Representative ESR spectra of DPPH radical. DPPH radical scavenging activity was calculated relative to control by quantifying the DPPH ESR signal intensity (A) under each condition. (a2) Relative DPPH signal intensities (mean ± SD, n = 3). Neither Q alone nor H_2_ alone significantly reduced the ESR signal intensity. However, the combination of Q and H_2_ nearly abolished the DPPH signal (≥99% scavenged), equivalent to the preformed QH_2_ positive control. These data strongly suggest *in situ* formation of QH_2_ or similar reduced intermediates through semiquinone radicals upon H_2_ addition. **(b)** HPLC quantification of QH_2_ produced in a KO_2_-driven chemical O_2_•^-^ generation system (50 μM Q, pH6.0) with or without 0.8 mM H_2_. Data from 27 replicate presented as box plots: box: interquartile range, horizontal line: median: marker: mean; whiskers 5th-95th percentile. Even without H_2_, approximately 3.7% of Q was spontaneously reduced to QH_2_, likely due to reaction with O_2_•^-^. Upon addition of H_2_, QH_2_ formation significantly increased to approximately 7.1% (paired t-test, *p* < 0.05), indicating H_2_-driven Q reduction. High replicate variability may reflect the stochastic nature of electron tunneling processes in the non-enzymatic experimental system.

As summarized in Figure 4a(1–2), ESR analysis showed minimal radical scavenging by either Q alone (∼0.4% signal reduction vs. control) or H_2_ alone (∼2.5% reduction), neither reaching statistical significance. However, when Q and H_2_ were simultaneously present, the ESR intensity of DPPH decreased drastically (≥99% reduction), a level comparable to the addition of authentic, pre-formed QH_2_. Quantitatively, relative ESR signal intensities of DPPH signals were as follows: control 100%; Q alone, 99.6% ± 7.8%; H_2_ alone 97.5% ± 7.2%; QH_2_ positive control, 0.2% ± 0.14%; and Q + H_2_, 0.1% ± 0.009%.

These data clearly indicate that substantial radical scavenging activity occurs exclusively when both Q and H_2_ coexist, strongly implying the *in situ* formation of QH_2_ or closely related reducing intermediates derived from semiquinone radicals. Given that QH_2_ is a highly potent radical scavenger, the ESR results provide indirect yet compelling evidence that H_2_ actively participates in redox cycling mediated by semiquinone radicals.

### Quantitative HPLC evidence for QH_2_ formation in H_2_-Driven redox cycling

To directly confirm the formation of QH_2_ in reactions involving H_2_, Q, and O_2_•^-^, we conducted quantitative HPLC analyses under defined conditions (Figure 4b). Even in the absence of H_2_, a small baseline amount of Q was spontaneously reduced to QH_2_, (approximately 3.7%), presumably due to reaction with O_2_•^-^ generated in the system. This baseline formation of QH_2_ was not attributed to reducing impurities, as purity analyses by HPLC confirmed the absence of detectable reduced contaminants, including QH_2_.

Upon addition of 0.8 mM H_2_ under identical conditions, the average yield of QH_2_ significantly increased to approximately 7.1% (paired *t*-test, *p* < 0.05, n = 27), nearly double the baseline level. This result clearly indicates that H_2_ actively participate in the reduction of Q to QH_2_, providing direct quantitative evidence supporting the previously proposed semiquinone radical-mediated redox cycling mechanism.

Notably, substantial variability in QH_2_ yields between replicates was observed, reflected by coefficient of variation approximately 69% in the presence of H_2_, and approximately 102% without H_2_. This variability exceeds typical experimental error and is likely attributable to the inherent probabilistic nature of electron transfer processes in our non-enzymatic, structurally unorganized system. In such system, effective electron transfer through tunneling depends on the stochastic occurrence of molecular collisions and favorable orientations within short, typically sub-nanometer distances (Gray and Winkler, 2005). Consequently, even slight fluctuations in molecular proximity, orientation, or thermal motions can affect the probability of successful tunneling events, resulting in significant experimental variability.

Despite this inherent variability, the reproducible and statistically significant increase in QH_2_ formation upon H_2_ addition strongly supports a tunneling-related, non-classical electron transfer mechanism. Specifically, the observed variability and kinetic profiles suggest that electron transfer may occur through both direct O_2_•^-^-mediated activation of H_2_ and indirect Q•^-^-mediated activation pathways. This dual mechanistic framework effectively explains the complex kinetic phenomena, including the coexistence of distinct bell-shaped and U-shaped kinetic behaviors observed under varying redox conditions (Figures 1–3).

In summary, these quantitative HPLC results reinforce a coherent mechanistic model wherein H_2_ undergoes catalytic metal-free activation trough distinct redox path.

### Semiquinone (Q•^-^) as a mediator for H_2_ activation

Our experimental results provide compelling evidence that Q•^-^ serve as central mediators in the electron transfer reactions involving H_2_. A remarkable finding is that simply altering the availability of Q and varying H_2_ concentrations enabled us to capture distinct kinetic behavior consistent with both Marcus normal and inverted electron transfer regions. These observation supports the applicability of Marcus theory, which was originally developed for radiation chemistry and condensed-phase electron transfers (Yamazaki et al., 1999; Maroz et al., 2009), to biologically relevant electron transfer involving H_2_.

Our kinetic analyses also revealed consistently higher values of the pre-exponential factor *A* in the Marcus equation when Q was present, suggesting enhanced electronic coupling or increased effective collision frequency in the Q-mediated pathway. These results are intuitive, because Q function as an electron shuttle or radical mediator, temporarily storing electrons derived from H_2_. This transient electron storage in the reactive Q•^-^ intermediate, effectively facilitates subsequent electron transfers to O_2_ by reducing the requirement of simultaneous collision and precise molecular orientation necessary for direct tunneling between Q•^-^ and H_2_.

ESR results (Figure 4a) provided indirect but compelling evidence supporting Q•^-^-mediated electron transfer. The simultaneous presence of Q and H_2_ under conditions generating O_2_•^-^ resulted in radical scavenging activity of DPPH signals comparable to that of authentic QH_2_, indicating that generated Q•^-^ mediate electron transfer from H_2_ to form QH_2_ or related reductant intermediates. Quantitative HPLC analysis further corroborated the role of H_2_ in this Q•^-^-mediated electron transfer process (Figure 4b). QH_2_ formation from Q under the coexistence of O_2_•^-^ and Q•^-^, markedly increased (approximately doubling) by adding H_2_, indicating the critical role of Q•^-^ as mediators facilitating H_2_-driven redox cycling.

Taken together, our findings support a coherent mechanistic model in which Q•^-^ function as versatile, catalytic metal-free intermediates for H_2_ activation, enabling distinct electron transfer pathways depending critically on the redox environment.

## Discussion

### General implications and limitations

All experiments in this study were conducted in membrane-less, solution-phase *in vitro* systems (XO/Hx and KO_2_). Accordingly, the mechanistic interpretations proposed here are framed as testable hypotheses, not established biological facts. Our data show that H_2_ modulates O_2_•^-^ kinetics and promotes Q reduction to QH_2_ *in vitro*, supported by AUC/initial-velocity statistics and orthogonal ESR/HPLC readouts. These findings establish the chemical feasibility of H_2_-driven Q redox cycling in-solution; the physiological relevance remains to be determined in mitochondria and cells. The schematic of mitochondrial Complex I is therefore presented only as a conceptual figure in Supplementary Material (Supplementary Figure S1).

### Biological relevance and future directions

While our in-solution model supports the feasibility of H_2_ participation in Q redox cycling, physiological relevance remains unproven. The data are consistent with, but do not prove, a tunneling-assisted route via Q•^-^. Within these constraints, the present study provides an in-solution experimental framework and testable, hypothesis-generating evidence to guide targeted *in vivo*/*in situ* validation for future investigations aimed at elucidating the biological principles underlying redox modulation by H_2_. Intriguingly, recent studies indicate that the biological effects of Q itself may also not always exhibit simple dose-dependent benefits. For instance, partial suppression of Q biosynthesis in model organisms has paradoxically been associated with lifespan extension, presumably due to moderate elevation of ROS acting as signaling molecules (Wang et al., 2024). Our observations of biphasic effects of H_2_ on ROS, depending on the availability of Q, aligns with this perspective, emphasizing the importance of precise redox balance rather than linear dose-response relationship. Future studies should employ respiratory-active isolated mitochondria and purified mitochondrial respiratory complexes I and III to directly assess how varying H_2_ concentrations influence both exogenous and endogenous Q/QH_2_ ratios and mitochondrial ROS generation, particularly O_2_•^-^, under physiologically and pathologically relevant conditions (e, g., hypoxia, pseudo-hypoxia, or succinate induced RET). Such experiments are currently ongoing in our laboratory and will provide further validating and expand the biological implications of tunneling-mediated redox regulation by H_2_.

Beyond these biological implications, the discovery of a metal-independent pathway for activating H_2_ also has significant potential for green chemistry and sustainable energy technologies. Industrial activation of H_2_ typically relies on rare metal catalysts (e.g., Pt, Ni, etc.) for H_2_ cleavage. While our findings suggest that organic radials intermediates such as Q•^-^ or O_2_•^-^, might offer alternative approaches for metal-free H_2_ activation under mild conditions. Although transient radicals like Q•^-^ themselves may not be directly practical for industrial applications due to their instability, this concept can inspire the development of stable organic radical-based biomimetic catalysts or redox-active polymers capable of facilitating tunneling electron transfer. Moreover, a two-step electron relay system analogous to the Q/QH_2_ couple may circumvent high activation barriers for direct H_2_ oxidation, offering potential advancements in electrochemical energy conversion and hydrogen storage technologies.

Collectively, these results not only deepen fundamentally our understanding of mitochondrial redox biology and biological phenomena but also offer potential for novel therapeutic strategies targeting oxidative stress-related mitochondrial dysfunction and for innovations in sustainable technology.

### Relation to porphyrin/heme-based mechanisms

Our membrane-less in-solution system does not contain heme proteins; therefore, the observed bell/U-shaped kinetics arise without porphyrin cofactors and are consistent with semiquinone-linked electron transfer. Nonetheless, porphyrin/heme-centered routes—including Fe-porphyrin redox activity toward H_2_ and CO_2_➝CO under hypoxia—remain plausible in biological settings (Jin et al., 2023) and may relate with quinone pathways in mitochondria. In particular, our findings on Q/Q•^-^/QH_2_ chemistry remind the Q-cycle in complex III, where the redox reactions between cytochrome *b*_L_ and semiquinone plays pivotal roles, as previously discussed (Ishibashi, 2019). When focusing the Q•^-^-mediated activation of H_2_ in complex III, investigating the electron transfer between Q•^-^ and heme in cytochrome bL will provide an important insight to understand the modulations of redox balance by H_2_ in complex III, which are beyond the scope of the present in-solution work.

## Study Limitations

### Scope of H_2_ concentrations

We examined the effects of H_2_ at four discrete concentrations (0, 0.2, 0.4, 0.8 mM), which clearly demonstrated bell-shaped and U-shaped kinetic profiles. However, finer resolution near the critical transition points would more precisely demonstrate the optimal H_2_ concentration and exact thresholds between Marcus normal and inverted kinetic regions. Future studies employing narrower increments and extending beyond 1 mM would help clarify these critical points and how they vary under different redox conditions.

### Potential enzyme or metal artifacts

The enzymatic system (Hx/XO) employed in this study include XO, which contains molybdenum cofactors and iron-sulfur clusters. While XO primarily produces O_2_•^-^ from substrate (Hx) oxidation under aerobic conditions, we cannot completely exclude the possibility of unintended interactions between H_2_ and these metallic cofactors. Although unlikely, if XO or trace metal contaminants exhibited hydrogenase-like activity, it might influence our results. Nevertheless, we observed similar Marcus-type kinetics and QH_2_ formation in strictly enzyme-free chemical systems (KO_2_), convincingly indicating that our proposed H_2_ activation mechanism is fundamentally metal-independent.

### Transient detection of Q•^-^

Direct detection of semiquinone radical (Q•^-^) in aqueous solutions was experimentally impractical due to their extremely short lifetime and their coexistence with O_2_•^-^. We therefore inferred the presence of Q•^-^ indirectly, through ESR-based DPPH radical scavenging assays and modulation of O_2_•^-^ generation. Although these indirect methods provided consistent evidence supporting our conclusions, direct spectroscopic detection would strengthen mechanistic interpretations. Future studies using advance high-temporal-resolution techniques, such as pulsed radiolysis or rapid freeze-quench EPR, could directly monitor transient Q•^-^ formation and clarify precise reaction intermediates involved in H_2_ activation.

### Variability in QH_2_ measurements

The observed substantial variability in measured QH_2_ yields (coefficient of variation up to 100% in controls), reflecting inherent stochastic nature of tunneling-mediated electron transfer processes in our structurally unorganized, non-enzymatic experimental system. Such variability complicates quantitative interpretation of the efficiency with which H_2_ drives Q reduction to form QH_2_. Future studies employing temperature-dependent analyses or kinetic isotope effects using deuterated hydrogen (D_2_) could further clarify this variability. Demonstrating diminished or temperature-independent kinetic isotope effects would provide clearer features for tunneling mechanisms underlying these reactions.

## Conclusion

In summary, under membrane-less in-solution conditions, H_2_ modulates Q•^-^ kinetics and supports Q reduction to QH_2_without catalytic metals or hydrogenases. The observed bell- and U-shaped profiles vary with Q and H_2_ in a consistent manner with the Marcus electron transfer framework and are compatible with a tunneling-assisted route involving Q•^-^. These data support the chemical feasibility of H_2_ driven Q redox cycling *in vitro*; *in-vivo* relevance remains to be determined, motivating validation in respiring mitochondria and cellular models.

## Data Availability

The original contributions presented in the study are included in the article/Supplementary Material, further inquiries can be directed to the corresponding author.
