# Correction: Superoxide- and semiquinone-linked activation of molecular hydrogen in metal-catalyst-free solution

**DOI:** 10.3389/fmolb.2025.1760628

**Published:** 2025-12-23

**Authors:** Frontiers Editorial Office

**Affiliations:** Frontiers Media SA, Lausanne, Switzerland

**Keywords:** molecular hydrogen, semiquinone, superoxide, quantum biology, quantum tunneling, H2 activation, reverse electron transport, mitochondrial complex I and III

In the abstract, the term “SQ radicals” was erroneously used instead of “semiquinone radicals (Q•^-^)”. This has been corrected to read:

“Here we show that under membrane-less in-solution conditions, H_2_ modulates O_2_•^-^ kinetics in ways consistent with a tunneling-assisted electron transfer involving semiquinone radicals (Q•^-^), without catalytic metals or hydrogenases”.

The original article has been updated.

