# Supplementary material for: Superoxide- and semiquinone-linked activation of molecular hydrogen in metal-catalyst-free solution
Source: Front Mol Biosci. 2025 Oct 21;12:1680812. doi: 10.3389/fmolb.2025.1680812 (PMC12582930; doi:10.3389/fmolb.2025.1680812)
Supplement: Supplementary file 1 [file DataSheet2.pdf]

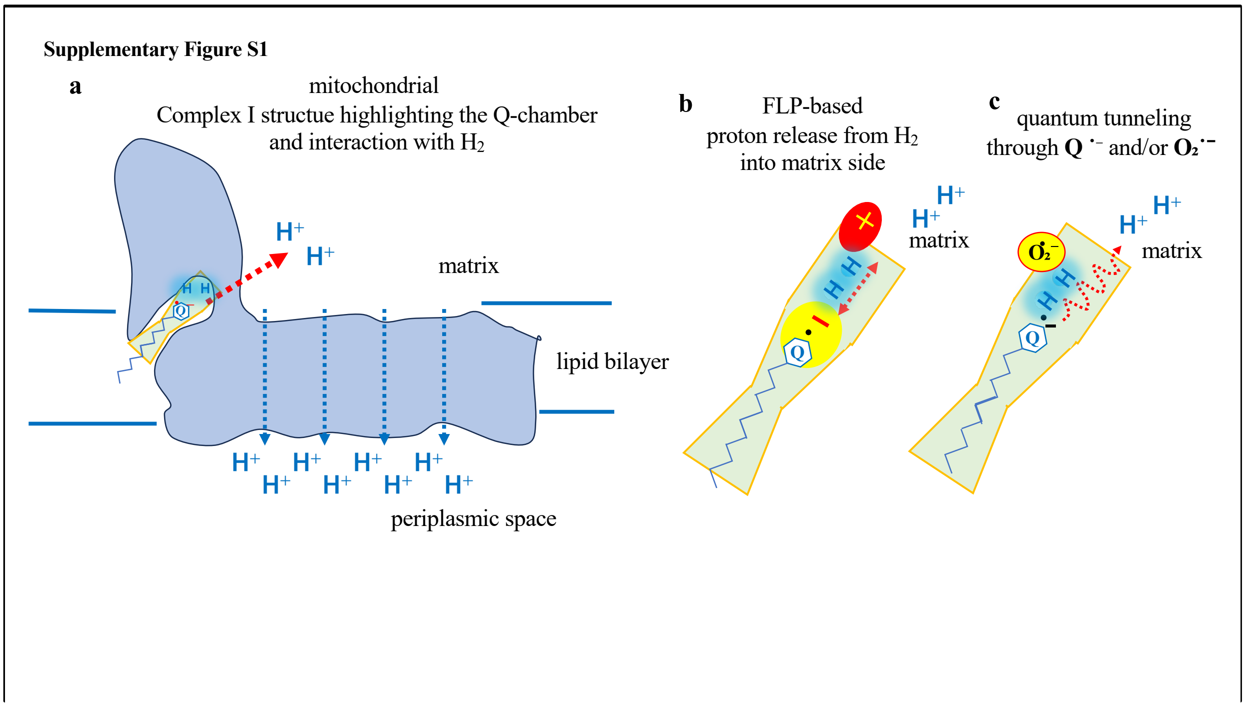

Figure 5. Hypothetical Schema: H_2 activation and proton release pathways within the Q-chamber in mitochondrial Complex I.
(a) Schematic representation of mitochondrial Complex I embedded within the inner mitochondrial membrane (lipid bilayer). The energy conversion from electron transfer results in a proton motive force with elevated mitochondrial membrane potential. The hydrophobic Q-chamber (pale green box with a bottle neck narrow lesion), located at the interface between hydrophilic matrix-side subunits and the membrane-embedded proton-pumping region, accommodates Q species. Here, $Q^{\bullet-}$ with their hydrophobic isoprenoid tails (zigzag lines), transiently accumulate and may interact directly with H_2. The protons derived from H_2 can be released from Q-chamber into the matrix (a red dashed arrows). This proton release modulates MMP, alleviating electron leakage during RET.
(b) Proposed metal-free frustrated Lewis pair (FLP)-like mechanism for H_2 activation within the Q-chamber (pale green box). $Q^{\bullet-}$ (in a yellow circle with negative charge) and basic amino acid residues (presented by a red oval with positive charge), such as His 55 or His 59 in mammalian Complex I, could polarize and weaken the H-H bond, facilitating proton release directly into the mitochondrial matrix [3].

(c) Proposed tunneling-assisted electron transfer mechanism for H_2 activation within the Q-chamber (pale green box). In this pathway, electron tunneling (indicated by a red dotted arrow) may occur between H_2 and $Q^{\bullet -}/O_2^{\bullet -}$. This tunneling-mediated activation can take place either independently or synergistically with the FLP-like catalytic activation shown in (b), providing a versatile mechanism for catalytic-metal independent H_2 activation within mitochondrial Complex I.
