# Supplementary material for: Superoxide- and semiquinone-linked activation of molecular hydrogen in metal-catalyst-free solution
Source: Front Mol Biosci. 2025 Oct 21;12:1680812. doi: 10.3389/fmolb.2025.1680812 (PMC12582930; doi:10.3389/fmolb.2025.1680812)
Supplement: Supplementary file 2 [file DataSheet1.pdf]

Supplementary Note

Supplementary Table S1 summarizes the parameters obtained from nonlinear fitting of AUC values to the Marcus rate expression across Q conditions. The reorganization energy (λ) ranged from 0.120 to 0.180 eV, consistent with efficient electron transfer in the Marcus normal region. Notably, Q = 0 µM yielded a bell-shaped response with λ = 0.147 eV, characteristic of single-path inverted region behavior, while Q = 10 and 50 µM produced U-shaped curves, supporting a multi-step electron transfer model via $Q^{\bullet-}$. The amplitude parameter (A) also increased with Q concentration, potentially reflecting enhanced redox buffering and semiquinone-mediated tunneling at higher Q levels.

Supplementary Table S1 Marcus Fit Parameters

Condition	Reorganization energy (λ)	Amplitude (A)	Curve shape
Q = 0 µM	0.147	1.019	Bell-shaped
Q = 10 µM	0.12	0.85	U-shaped
Q = 50 µM	0.18	1.05	U-shaped

Supplement Eq. S2 (Q-free barrier).

Using $E^0_{O_2/O_2^{\bullet-}}$ = -0.16 V and $E^0_{H_2/H^+}$ = -0.41 V, the driving force per electron is ΔG^0 = -(E^0_{acc} - E^0_{don}) = -(-0.16-(-0.41)) = 0.25 eV. With the fitted reorganization energy λ = 0.147 eV, the Marcus barrier is

$$\Delta G^{\ddagger} = \frac{(\Delta G^0 + \lambda)^2}{4\lambda} = \frac{(-0.25+0.147)^2}{4x0.147} = 0.017\text{-}0.018 \text{ eV}$$

Supplementary Tables

Table S2a | Statistical analysis of $O_2^{\bullet -}$ generation rates (Hx = 2 μM). Two-way ANOVA assessing effects of quinone (Q) and molecular hydrogen (H_2) concentrations, and their interaction. Grey shading indicates significant differences ($p < 0.05$).

One-way ANOVA for Q effects per H_2 concentrations			One-way ANOVA for H_2 effects per Q concentrations			Two-way ANOVA		
Q (μM)	F-value	p-value	H_2 (mM)	F-value	p-value		F-value	p-value
0	133	3.35×10^{-46}	0	125.6	3.80×10^{-44}	Q (μM)	107	4.73×10^{-44}
10	89.1	2.27×10^{-33}	0.2	102	2.25×10^{-37}	H_2 (mM)	53.3	4.91×10^{-23}
50	92.1	2.64×10^{-34}	0.8	112	2.40×10^{-40}	Q x H_2 interaction	120	4.29×10^{-88}

Table S2b | Statistical analysis of $O_2^{\bullet -}$ generation rates (Hx = 5 μM). Two-way ANOVA assessing effects of quinone (Q) and molecular hydrogen (H_2) concentrations, and their interaction. Grey shading indicates significant differences ($p < 0.05$).

One-way ANOVA for Q effects per H_2 concentrations			One-way ANOVA for H_2 effects per Q concentrations			Two-way ANOVA		
Q (μM)	F-value	p-value	H_2 (mM)	F-value	p-value		F-value	p-value
0	132	4.24×10^{-46}	0	236	4.97×10^{-71}	Q (μM)	142	9.58×10^{-57}
10	156	2.62×10^{-52}	0.2	41.0	4.04×10^{-17}	H_2 (mM)	3.54	0.0293
50	6.56	0.00160	0.8	222	4.30×10^{-68}	Q x H_2 interaction	94.0	1.09×10^{-70}

Table S3a | Statistical analysis of AUC at Hx = 2 μM. Two-way ANOVA evaluating main effects and interaction between Q and H_2. Grey shading denotes significant differences ($p < 0.05$).

One-way ANOVA for Q effects per H_2 concentrations			One-way ANOVA for H_2 effects per Q concentrations			Two-way ANOVA		
Q (μM)	F-value	*p*-value	H_2 (mM)	F-value	*p*-value		F-value	*p*-value
0	18.0	0.000710	0	138	1.75×10^{-7}	Q (μM)	57.0	2.04×10^{-10}
10	68.6	3.57×10^{-6}	0.2	13.8	0.00182	H_2 (mM)	28.2	2.46×10^{-7}
50	192	4.12×10^{-8}	0.8	187	4.73×10^{-8}	Q x H_2 interaction	63.5	2.43×10^{-13}

Table S3b | Statistical analysis of AUC at Hx = 5 μM. Two-way ANOVA evaluating main effects and interaction between Q and H_2. Grey shading denotes significant differences ($p < 0.05$).

One-way ANOVA for Q effects per H_2 concentrations			One-way ANOVA for H_2 effects per Q concentrations			Two-way ANOVA		
Q (μM)	F-value	*p*-value	H_2 (mM)	F-value	*p*-value		F-value	*p*-value
0	325	4.09×10^{-9}	0	298	5.95×10^{-9}	Q (μM)	47.0	1.61×10^{-9}
10	219	2.35×10^{-8}	0.2	12.3	0.00264	H_2 (mM)	1.29	0.292
50	1.52	0.270	0.8	54.3	9.46×10^{-6}	Q x H_2 interaction	31.7	7.60×10^{-10}

Table S4 | Statistical analysis of initial reaction velocities (1–3 min) at Hx = 200 μM. Analysis highlighting significant nonlinear interactions among Q, H_2, and $O_2^{\bullet-}$ conditions. Grey shading indicates significant results ($p < 0.05$).

One-way ANOVA for Q effects per H_2 concentrations			One-way ANOVA for H_2 effects per Q concentrations			Two-way ANOVA		
Q (μM)	F-value	*p*-value	H_2 (mM)	F-value	*p*-value		F-value	*p*-value
0	11.6	0.000735	0	86.9	1.30×10^{-6}	Q (μM)	333	6.02×10^{-24}
100	6.58	0.00705	0.2	271	9.10×10^{-9}	H_2 (mM)	9.38	0.000102
250	1.90	0.184	0.4	60.9	5.87×10^{-6}	Q x H_2 interaction	9.70	2.33×10^{-6}
			0.8	68.9	3.50×10^{-6}			

Table S5a | Statistical analysis of AUC under moderate oxidative load (KO_2 = 1 mM). Two-way ANOVA evaluating main effects and interactions between Q and H_2. Grey shading indicates statistical significance (p < 0.05).

One-way ANOVA for Q effects per H_2 concentrations			One-way ANOVA for H_2 effects per Q concentrations			Two-way ANOVA		
Q (μM)	F-value	*p*-value	H_2 (mM)	F-value	*p*-value		F-value	*p*-value
0	4.46	0.0452	0	20.3	0.000054	Q (μM)	10.6	0.00004
5	17.0	0.000870	0.2	0.637	0.605	H_2 (mM)	9.01	0.000672
10	3.49	0.0757	0.8	27.4	0.000012	Q x H_2 interaction	5.50	0.000401
50	6.14	0.0208						

Table S5b | Statistical analysis of AUC under high oxidative load (KO_2 = 2.5 mM). Two-way ANOVA evaluating main effects and interactions between Q and H_2. Grey shading indicates statistical significance (p < 0.05).

One-way ANOVA for Q effects per H_2 concentrations			One-way ANOVA for H_2 effects per Q concentrations			Two-way ANOVA		
Q (μM)	F-value	*p*-value	H_2 (mM)	F-value	*p*-value		F-value	*p*-value
0	0.271	0.769	0	42.1	0.000001	Q (μM)	79.7	5.79×10^{-16}
5	3.89	0.0605	0.2	11.4	0.000785	H_2 (mM)	1.14	0.330
10	0.289	0.756	0.8	80.2	3.29×10^{-8}	Q x H_2 interaction	2.18	0.0678
50	4.91	0.0361						
